# Restrictive Cardiomyopathy Caused by Troponin Mutations: Application of Disease Animal Models in Translational Studies

**DOI:** 10.3389/fphys.2016.00629

**Published:** 2016-12-19

**Authors:** Xiaoyan Liu, Lei Zhang, Daniel Pacciulli, Jianquan Zhao, Changlong Nan, Wen Shen, Junjun Quan, Jie Tian, Xupei Huang

**Affiliations:** ^1^Cardiovascular Research Laboratory, Division of Cardiology, Chongqing Medical University Children's HospitalChongqing, China; ^2^Department of Biomedical Science, Charles E. Schmidt College of Medicine, Florida Atlantic UniversityBoca Raton, FL, USA; ^3^Department of Cardiology, Bayannaoer City HospitalBayannaoer, China

**Keywords:** myofibrils, troponin, mutation, cardiomyopathy, diastolic dysfunction, animal models

## Abstract

Cardiac troponin I (cTnI) plays a critical role in regulation of cardiac function. Studies have shown that the deficiency of cTnI or mutations in cTnI (particularly in the C-terminus of cTnI) results in diastolic dysfunction (impaired relaxation) due to an increased myofibril sensitivity to calcium. The first clinical study revealing the association between restrictive cardiomyopathy (RCM) with cardiac troponin mutations was reported in 2003. In order to illustrate the mechanisms underlying the cTnI mutation caused cardiomyopathy, we have generated a cTnI gene knockout mouse model and transgenic mouse lines with the reported point mutations in cTnI C-terminus. In this paper, we summarize our studies using these animal models from our laboratory and the other *in vitro* studies using reconstituted filament and cultured cells. The potential mechanisms underlying diastolic dysfunction and heart failure caused by these cTnI C-terminal mutations are discussed as well. Furthermore, calcium desensitizing in correction of impaired relaxation in myocardial cells due to cTnI mutations is discussed. Finally, we describe a model of translational study, i.e., from bedside to bench and from bench to bedside. These studies may enrich our understanding of the mechanism underlying inherited cardiomyopathies and provide the clues to search for target-oriented medication aiming at the treatment of diastolic dysfunction and heart failure.

## Introduction

Cardiac cells (myocardium) consist of two filaments: Thick filament and thin filament. The former contains mainly myosin and myosin bind C-protein and the latter contains actin and troponin-tropomyosin complex. The so called cross-bridge formation between the thin filament actin and the thick filament myosin determines the filament movement, i.e., muscle contraction and relaxation. Whereas, the troponin complex plays an important role in regulation of the filament movement. Troponin complex consists of three subunit proteins: Troponin C (TnC), troponin T (TnT), and troponin I (TnI). Among them, TnC is a Ca^2+^ binding protein, TnT binds with tropomyosin whereas TnI is an inhibitory subunit that can bind to actin-tropomyosin and prevent muscle contraction by inhibition of actin-tropomyosin- activated myosin (actomyosin) ATPase activity (Greaser et al., 1972; Ohtsuki and Shiraishi, 2002). Many studies have demonstrated that TnI has an important function in the regulation of striated muscle contraction and relaxation (Kranias and Solaro, 1982; El-Saleh et al., 1986; Zot and Potter, 1987; Solaro and Troponin, 1999; Konhilas et al., 2003).

It is well-known that cardiac muscle movement, i.e., contraction and relaxation, is regulated mainly by intracellular calcium. An increase of intracellular Ca^2+^ concentration results in an enhanced cardiac contractility whereas the decreased Ca^2+^ concentration can reduce the cardiac contractility. The concentration of intracellular Ca^2+^ is regulated by various calcium handling proteins in myocardium, such as Ca^2+^ channel receptors, the SERCA2a calcium ATPase pump, phospholamban, etc. (Gordon et al., 2000). Recently, a body of studies has demonstrated that cardiac TnI (cTnI) has unique functions in control of cardiac muscle contraction and relaxation, especially in diastolic function (Yasuda et al., 2007; Solaro et al., 2008). PKA-mediated cTnI phosphorylation causes a decrease of myofibril sensitivity to Ca^2+^ or a desensitization of the contractile apparatus to activation by Ca^2+^ (Solaro et al., 1976; Rorbertson et al., 1982; Zhang et al., 1995; Chandra et al., 1997; Solaro, 2001; Metzger and Westfall, 2004; Periasamy and Janssen, 2008). Using a cTnI gene knockout mouse model that generated by Huang et al in 1999, we have demonstrated that impaired relaxation occurs in myocardial cells with a deficiency of TnI and sarcomere length from these cells is shortened due to an increased tension even in the absence of calcium, suggesting that cardiac TnI is critical for cardiac relaxation (Huang et al., 1999).

Physiologically, TnI plays such a critical role in regulation of cardiac function, especially the muscle relaxation. In the following sections, we will discuss the cardiac dysfunction caused by cTnI C-terminal structural changes, i.e., cTnI mutations, in the heart under pathological conditions. In addition, the potential mechanisms underlying the diastolic dysfunction are discussed as well.

## *In vitro* assays measuring the effects of cTnI mutations on myofibril function

Many studies have confirmed that the C-terminal half of cTnI is more conserved than the N-terminal region of the protein (Wilkinson and Grand, 1978). The C-terminal part of cTnI contains specific regions that are crucial for the normal activity of the protein, in particular, cardiac relaxation. In a part of cTnI, there is an inhibitory region that is the minimum sequence necessary for inhibition of actomyosin ATPase activity. This domain includes residues from 147 to 163 and binds strongly to actin and the N terminal domain of TnC regulating the binding of Ca^2+^ to TnC (Rieck and Dong, 2014). The region between the residues from 168 to 188 in cTnI is a second actin-binding site that binds specifically to the actin-tropomyosin filament and is known to contribute to the inhibitory activity of cTnI (Tripet et al., 1997). The remaining C-terminal domain, 192 to 210 is not fully characterized, however, some studies indicate that this part of cTnI plays a role in the stabilization of tropomyosin in the actin filament upon Ca^2+^ activation (Galińska et al., 2010).

The integrity of the cTnI molecule is essential for proper conformation of the troponin complex in the myofilament and the inhibition of actomyosin ATPase activity. It is of great importance, both scientifically and clinically, to elucidate the cellular mechanisms underlying RCM caused by cTnI mutations in order to identify the cause of cardiomyopathies and heart failure. The data from analyzing *in vitro* reconstituted thin filaments showed that the RCM cTnI mutations had high Ca^2+^-sensitizing effects on cardiac muscle force generation (Gomes et al., 2005; Kobayashi and Solaro, 2006). The reconstituted filament assays have the advantage of easily obtaining the mutated proteins and quickly testing the myofilament force generations. Very recently, this technique has been applied to explore the role of cardiac troponin I C-terminal mobile domain and linker sequence in regulating cardiac contraction (Meyer and Chase, 2016). Some research groups investigated the role of the mutated troponin in intact cells. Using an acute genetic engineering technique, Davis et al. transferred the mutant cTnI genes into cultured rat myocardial cells and found that the myofibril sensitivity to calcium was increased (Davis et al., 2007, 2008). Numerous mutations in the carboxyl half of the protein are associated with the development of cardiomyopathies further confirming the importance of the C terminal domains of cTnI for proper regulation of cardiac contraction (Chang et al., 2008; Tachampa et al., 2008). Drastic Ca^2+^ sensitivity change has been reported in myofilament with cTnI K178E mutation (Yumoto et al., 2005). However, most of the RCM mutations in cTnI have not been incorporated into transgenic models and they have been just characterized in functional *in vitro* studies.

## Cardiomyopathies caused by cTnI mutations: translational studies

Cardiomyopathies have been considered to represent diseases that primarily affect cardiac muscle. Based on their morphology and pathophysiology, three major types of cardiomyopathies are most prevalent: Hypertrophic cardiomyopathy (HCM), dilated cardiomyopathy (DCM), and restrictive cardiomyopathy (RCM) (Rivenes et al., 2000). HCM is characterized by a hypertrophic heart and DCM is characterized by a dilated ventricle, which are relatively easier to be recognized clinically. However, RCM, unlike HCM and DCM, manifests itself as a restricted ventricle that prevents or reduces the blood return to the heart because of a stiffened ventricle (Rivenes et al., 2000). Among the three major types of cardiomyopathies, RCM cases are not as common as HCM or DCM, but the prognosis is poor and some RCM patients die in their childhood (Rivenes et al., 2000; Palka et al., 2003). The clinical features of RCM are described as biatrial dilation, along with normal left ventricular internal dimension characterized on echocardiography. A marked elevation of left ventricular end-diastolic pressure with a restricted left ventricular filling and decreased cardiac output are often observed in RCM patients (Ligi et al., 2003; Palka et al., 2003). In the past, most cardiomyopathy cases were described as idiopathic cardiomyopathies, i.e., etiology is unknown (Ammash et al., 2000; Ligi et al., 2003). Recently with the advancement of genetic and molecular biological techniques, we know that most cardiomyopathy cases are heritable and caused by a single gene mutation (Braunwald, 2008).

The first report on cTnI C-terminal mutations associated human restrictive cardiomyopathy (RCM) was in 2003 (Mogensen et al., 2003). In that study, six cTnI mutations (L144Q, R145W, A171T, K178E, D190G, and R192H) have been found to be associated with RCM. Among them, the two mutations K178E and R192H have the worst clinical phenotype (Mogensen et al., 2003).

Our laboratory has participated in the studies to define the effect of the troponin mutations on the development of diastolic dysfunction. We have generated transgenic (TG) mice (cTnI^193His^) modeling human RCM mutation cTnI R192H (cTnI R193H in mouse sequence) in the heart. In addition, our laboratory has created another TG mouse line containing the RCM cTnI K178E mutation reported by Mogensen et al. (2003). The transgenic animals (cTnI K179E in the mouse genome) presented drastic bi-atrial enlargement in the absence of ventricular hypertrophy and dilation. They presented similar hemodynamic characteristics to the cTnI^193His^ animals in our laboratory confirming the development of RCM as a consequence of cTnI mutation. The cardiac dysfunction was severe in the animals as most of them died prematurely (Jean-Charles et al., 2008). The drastic hypersensitivity to Ca^2+^ observed in myocardium from our transgenic animal models is very similar to that reported from the *in vitro* studies (Yumoto et al., 2005).

We have tried to understand the mechanisms underlying the development of RCM due to cTnI mutations using the transgenic mice (cTnI^193His^) expressing human RCM mutation cTnI R192H (cTnI R193H in mouse sequence) in the heart. Histological examination confirms that cTnI^193His^ mice do not show cardiac hypertrophy or ventricular dilation. The general morphology of the ventricles from these mice is similar to that of a wild type heart. However, the enlargement of bi-atria, both right and left atria, is very dramatic, which is similar to that in human RCM patients carrying cTnI R192H mutation. Functional measurements on these mice indicate a diastolic dysfunction in the early stage and a diastolic heart failure in the late stage (Du et al., 2006). We have demonstrated that impaired relaxation is a main manifestation in the RCM cTnI transgenic mice (Du et al., 2008) and cTnI mutation caused myofibril Ca^2+^ hypersensitivity is a key factor resulting in a delayed calcium dissociation from the myofilaments and a delayed relaxation time (Li et al., 2010).

Using this animal model of disease, we have performed a series of cell-based experiments to determine diastolic dysfunction and calcium dynamics at a single myocardial cell level. Meanwhile, we have tried to reveal the cellular mechanisms of myofilament dysfunction in myocardial cells isolated from RCM mouse heart with cTnI mutations. Furthermore, we have measured left ventricular pressure using a Millar catheter in RCM mice to demonstrate that the increased pressure in restricted ventricles is due to increased internal tension in the wall of the ventricles caused by the myofibril hypersensitivity to Ca^2+^ (Zhang et al., 2015; Wang et al., 2016). Once we recognized that Ca^2+^ hypersensitivity was an important factor that is associated with impaired relaxation in myofibril cells resulting in a diastolic dysfunction in RCM mice with cTnI mutations, we have tried to reduce the hypersensitivity to calcium and hoped to reverse the phenotype in RCM mice. By crossing our cTnI^193His^ RCM mice with another transgenic mouse line (cTnI-ND) that expresses the cTnI with N-terminal deleted in the heart, we discovered that the hyposensitivity caused by cTnI-ND favored a general balance of myofibril sensitivity to calcium in the heart and reversed the diastolic dysfunction and rescued RCM phenotype (Li et al., 2010). Our study has demonstrated that desensitization of myofibrils to calcium can be a therapeutic target for restrictive cardiomyopathy with diastolic dysfunction. Later, another study using a different mouse line also confirmed that reduction of myofibril sensitivity to calcium was able to correct diastolic dysfunction in mice suffering from HCM (Alves et al., 2014).

Another similar example of cTnI C-terminal mutation-associated diastolic dysfunction and hypersensitivity to Ca^2+^ is cTnI R145W mutation. The mutation of cTnI R145W associated human RCM is first reported by Mogensen (Mogensen et al., 2003). Transgenic mice modeling human cTnI R145W was generated. Characterization of these cTnI R145W transgenic mice (Tg-R145W) has shown that the Tg-R145W myofibers have a large increase in the Ca^2+^ sensitivity of both force development and ATPase (Wen et al., 2009). Recent study using the recombinant human cardiac sarcomeres containing cTnI R145W mutation confirms that cTnI R145W mutation induces an increase in myofilament Ca^2+^ sensitivity by reducing the interaction between Helix-C of cTnC and cTnI (Dvornikov et al., 2016).

Increased Ca^2+^ sensitivity in myofilaments with cTnI C-terminal mutations is a key feature in cardiac muscle pathology. Therefore, it is urgent and necessary to search and find Ca^2+^ desensitizers that primarily affect myofilament sensitivity to Ca^2+^. So far, compounds with such properties are very scarce. Myosin inhibitors such as blebbistatin and 2, 3-butanedione monoxime (BDM) may alter myofilament sensitivity to Ca^2+^ via their inhibitory effect on actomyosin cross-bridge formation (Gwathmey et al., 1991; Kettlewell et al., 2004). These myosin ATPase inhibitors, while useful in functional studies *in vitro* and *ex vivo*, are too toxic for therapeutic use in live experimental animals or humans (Gwathmey et al., 1991; Kettlewell et al., 2004; Dou et al., 2007). There is a great need to develop or find small molecules and chemical Ca^2+^ desensitizers that can be used to alter myofibril sensitivity for Ca^2+^. Biological agents should be non-toxicity and have good bioavailability, but there are few Ca^2+^ desensitizers possessing such qualities. The catechin, (-)-epigallocatechin-3-gallate (EGCg) has Ca^2+^ desensitizing abilities via its interaction with cTnC (Liou et al., 2008; Robertson et al., 2009). This compound is the most abundant catechin in green tea and is credited for the numerous health benefits attributed to green tea consumption (Robertson et al., 2009). EGCg desensitizes thin filaments to Ca^2+^ by forming a ternary complex with the C-terminal domain of troponin C and the anchoring region of cTnI (Liou et al., 2008). The affinity of TnC for Ca^2+^ is reduced as a result which facilitates cardiac relaxation (Liou et al., 2008). The ability of EGCg to correct myofilament Ca^2+^ hypersensitivity and diastolic dysfunction has been demonstrated in a HCM mouse model confirming the therapeutic potential of that compound for diastolic dysfunction (Tadano et al., 2010).

**Figure 1:**
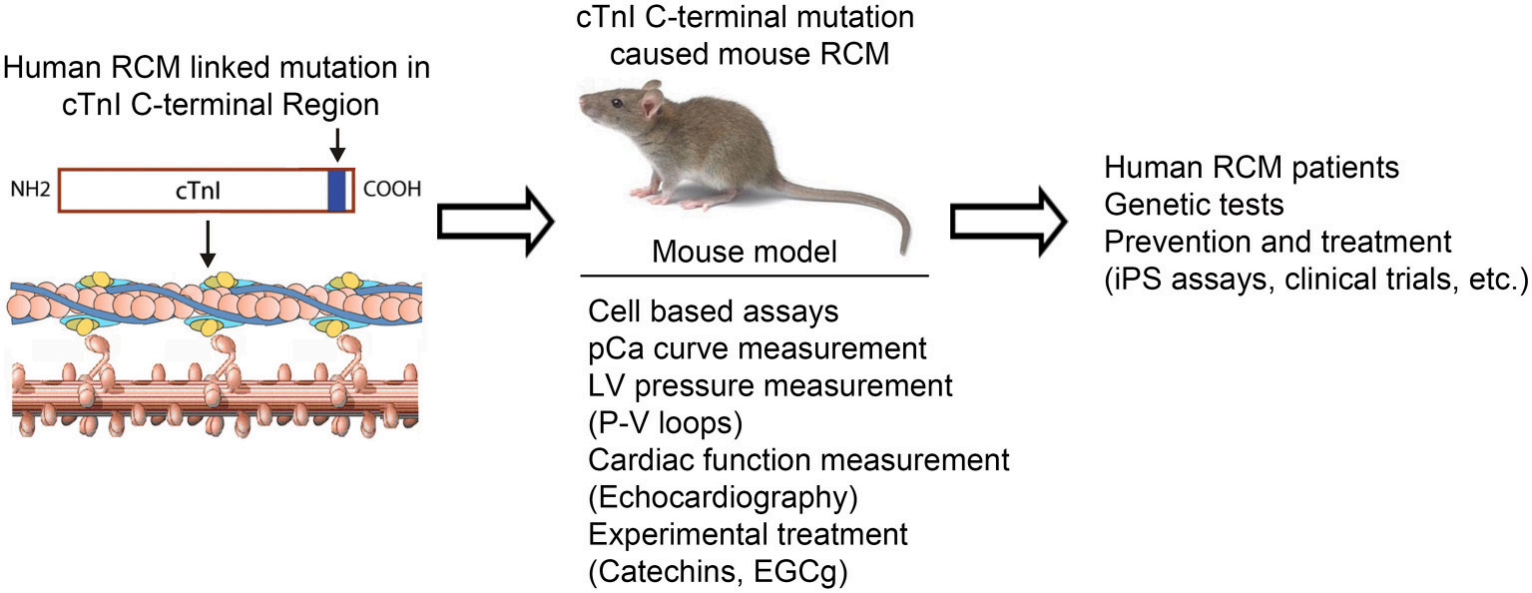
**Model of translational study using transgenic mice as tools to investigate the mechanism of the cTnI mutation caused RCM and to be used for experimental treatment**.

In our recent study, we have reported that diastolic dysfunction is corrected in RCM mice after the treatment of EGCg for 3months, suggesting that desensitizer catechin extracted from green tea is helpful in correcting impaired relaxation caused by calcium hypersensitivity in cTnI^193His^ RCM mice (Li et al., 2010). After our study, another group reported that green tea catechin could normalize the enhanced calcium sensitivity of myofilaments regulated by a HCM-associated mutation in human patient (Warren et al., 2015). These data confirm that desensitizing green extract catechin is able to reduce the hypersensitivity caused by cTnI mutations and correct the diastolic dysfunction. So far, we have received these cell-based and organ-based data from our studies using transgenic mouse models. It is difficult to obtain these data from human patient studies. This is a good model of translational study from bedside to bench as illustrated in Figure 1. The idea is that the physicians receive the disease information from the patients and the basic researchers use the information to create animal models of disease to confirm the clinical discovery and use the animal models to further explore the cellular and molecular mechanisms underlying the disorders. The data from basic research can provide information back to clinical studies and the treatment of the disease. For example, the data we have obtained so far could provide us with some clues for future clinical studies and disease treatment. Recently, we have collected samples from RCM patients in an out-patient department at a Children's Hospital in China. The data from genetic tests confirm that among five RCM patients, two patients carry 192 point mutation in cTnI gene and two carry a point mutation in myosin gene, and one patient with no detectable myofibril protein mutation. More experimental treatment data will be collected in this study from more RCM patients.

## Conclusions

Cardiac troponin plays a critical role in cardiac contraction and relaxation. cTnI deficiency or mutations are associated with RCM characterized with a diastolic dysfunction. The discovery of sarcomeric protein mutations responsible for the development of the disease helps in identifying the etiology of RCM and allows for the screening of potential RCM patients. These measures may facilitate early diagnostic of the disease and proper monitoring and management of RCM patients. It also paves the way for the development of transgenic animals with the RCM phenotype which will contribute greatly to a better understanding and characterization of the disease. In fact, RCM transgenic animals may provide a link in the translational study which is from bedside to bench and from bench to bedside. They will also be very useful for the trial of potential drugs or devices designed to correct the diastolic dysfunction associated with RCM. The lack of effective treatments and the unavailability of drugs that selectively correct the diastolic dysfunction of the restricted heart, make the development of new pharmacological agents an urgent necessity. Desensitizing green tea extract catechin has been proved to be useful in correcting hypersensitivity and reversing diastolic dysfunction both in RCM animal studies and in reconstitute myofilament assays using a cTnI with a point mutation from HCM patient. It seems promising to apply desensitizing green tea extract catechin in correcting impaired relaxation in RCM patients caused by troponin mutations.

## Author contributions

XL, DP, JZ, CN, WS, and XH participated in paper writing and LZ, JQ, and JT participated in patient sample collection and genotyping measurements.

### Conflict of interest statement

The authors declare that the research was conducted in the absence of any commercial or financial relationships that could be construed as a potential conflict of interest.
